# Supplementary material for: Plasma brain-derived tau is an amyloid-associated neurodegeneration biomarker in Alzheimer’s disease
Source: Nat Commun. 2024 Apr 4;15:2908. doi: 10.1038/s41467-024-47286-5 (PMC10995141; doi:10.1038/s41467-024-47286-5)
Supplement: Supplementary file 1 — Supplementary Information [file 41467_2024_47286_MOESM1_ESM.pdf]

Plasma brain-derived tau is an amyloid-associated neurodegeneration biomarker in Alzheimer's disease

Fernando Gonzalez-Ortiz[1,2]*; Bjørn-Eivind Kirsebom[3,4,5]*; José Contador[6,7,8]; Jordan E. Tanley[9]; Per Selnes[10,]; Berglind Gísladóttir[10]; Lene Pålhaugen[10]; Mathilde Suhr Hemminghyth[11,12,13]; Jonas Jarholm[10]; Ragnhild Skogseth[14,15]; Geir Bråthen[10]; Gøril Grøndtvedt[16,17]; Atle Bjørnerud[18,19,20]; Sandra Tecelao[10]; Knut Waterloo[10]; Dag Aarsland[21,22]; Aida Fernández-Lebrero[,6,7,8,23,24]; Greta García-Escobar[7,24]; Irene Navalpotro-Gómez[6,7,8,24]; Michael Turton[25]; Agnes Hesthamar[1]; Przemyslaw R. Kac[1]; Johanna Nilsson[1]; Jose Luchsinger[9]; Kathleen M. Hayden[9]; Peter Harrison[25]; Albert Puig-Pijoan[6,7,24,26]; Henrik Zetterberg[1,2,27,28,29,30]; Timothy M Hughes[9]; Marc Suárez-Calvet[6,7,8,31]; Thomas K. Karikari[1,32]**; Tormod Fladby[10,33]**; Kaj Blennow[1,2]**

1. Department of Psychiatry and Neurochemistry, Institute of Neuroscience and Physiology, the Sahlgrenska Academy at the University of Gothenburg, Mölndal, Sweden
2. Clinical Neurochemistry Laboratory, Sahlgrenska University Hospital, Mölndal, Sweden
3. Department of Neurology, University Hospital of North Norway, Tromsø, Norway
4. Department of Psychology, Faculty of Health Sciences, The Arctic University of Norway, Tromsø, Norway
5. Department of Neurology, Akershus University Hospital, Lørenskog, Norway
6. Barcelonaβeta Brain Research Center (BBRC), Pasqual Maragall Foundation, Barcelona, Spain
7. Hospital del Mar Research Institute, Barcelona, Spain
8. Cognitive Decline and Movement Disorders Unit, Neurology Department, Hospital del Mar, Barcelona, Spain.
9. Department of Internal Medicine, Section on Gerontology and Geriatric Medicine, Wake Forest University School of Medicine, Winston-Salem, North Carolina
10. Department of Neurology, Akershus University Hospital, Lørenskog, Norway
11. Research Group for Age-Related Medicine, Haugesund Hospital, Haugesund, Norway
12. Department of Neuropsychology, Haugesund Hospital, Haugesund, Norway
13. Department of Clinical Medicine (K1), University of Bergen, Bergen, Norway
14. Department of Geriatric Medicine, Haraldsplass Deaconess Hospital, Bergen, Norway.
15. Department of Clinical Sciences, Faculty of Medicine, University of Bergen, Norway.
16. Department of Neurology and Clinical Neurophysiology, University Hospital of Trondheim, Trondheim, Norway
17. Department of Neuromedicine and Movement Science, Faculty of Medicine and Health Sciences, Norwegian University of Science and Technology, Trondheim, Norway
18. Department of Physics, University of Oslo, Oslo, Norway.
19. Unit for Computational Radiology and Artificial Intelligence, Oslo University hospital, Oslo, Norway.
20. Department of Psychology, Faculty for Social Sciences, University of Oslo, Oslo, Norway.
21. Department of Old Age Psychiatry. Institute of psychiatry, Psychology and Neuroscience King's College London UK
22. Centre for Age-Related Diseases. Stavanger University Hospital Stavanger, Norway
23. Department of Medicine and Life Sciences, Universitat Pompeu Fabra, Barcelona (08003), Spain.
24. ERA-Net on Cardiovascular Diseases (ERA-CVD) consortium, Barcelona, Spain.
25. Bioventix Plc, 7 Romans Business Park, East Street, Farnham, Surrey GU9 7SX, UK
26. Department of Medicine, Universitat Autònoma de Barcelona, Barcelona, Spain
27. Department of Neurodegenerative Disease, UCL Institute of Neurology, Queen Square, London, UK
28. UK Dementia Research Institute at UCL, London, UK
29. Hong Kong Center for Neurodegenerative Diseases, Clear Water Bay, Hong Kong, China
30. Wisconsin Alzheimer's Disease Research Center, University of Wisconsin School of Medicine and Public Health, University of Wisconsin-Madison, Madison, WI, USA
31. Centro de Investigación Biomédica en Red de Fragilidad y Envejecimiento Saludable (CIBERFES), Madrid, Spain
32. Department of Psychiatry, University of Pittsburgh, Pittsburgh, PA, USA
33. Institute of Clinical Medicine, Campus Ahus, University of Oslo, Norway

* These authors contributed equally

** These authors jointly supervised this work

Correspondence to: Fernando Gonzalez Ortiz

Fernando.gonzalez.ortiz@gu.se

Supplementary Table 1.-Characteristics of the MESA-MIND pilot cohort (cohort-4)

	**Total (N=305)**	
**Age** Mean (SD)	72.9 (7.4)	
**Female** n (%)	168 (55.1%)	
**Cognitively Normal** n (%)	203 (66.6%)	
**MCI** n (%)	76 (24.9%)	
**Dementia** n (%)	12 (3.9%)	
**MoCA** Mean (SD)	22.7 (4.2)	
**CDR** Median (IQR)	0 (0)	
APOE-ε4+ n (%)	89 (29.2%)	
**Race**		
**White** n (%)	151 (49.5%)	
**Black** n (%)	154 (50.5%)	
**Diabetes Mellitus**		
**Normal** n (%)	158 (52.1%)	
**Untreated Diabetes** n (%)	8 (2.6%)	
**Treated Diabetes** n (%)	59 (19.5%)	
**LDL** Mean (SD)	110.6 (36.3)	
**HDL** Mean (SD)	58.6 (16.2)	
**Total Cholesterol** Mean (SD)	187.9 (42.7)	
**BMI** Mean (SD)	28.8 (5.3)	
**eGFR** Mean (SD)	76.4 (20.6)	
	Mean (SD)	Median (IQR)
**BD-tau** Mean (SD), Median (IQR)	27.8 (85.9)	7 (3.8)
**NfL** Mean (SD), Median (IQR)	36.3 (35.9)	27.1 (20.6)
**Aβ40** Mean (SD), Median (IQR)	103.5 (28.8)	100 (26.8)
**Aβ42** Mean (SD), Median (IQR)	7.4 (2.2)	7.3 (2.5)
**GFAP** Mean (SD), Median (IQR)	127.6 (102.5)	100.5 (82.3)
**p-tau181** Mean (SD), Median (IQR)	17.1 (160.6)	6 (5.6)
**p-tau231** Mean (SD), Median (IQR)	18.1 (55.6)	13.3 (7.8)

Abbreviations: BMI = body mass index; HDL = high-density lipoprotein; LDL = low-density lipoprotein; eGFR = estimated glomerular filtration. Only descriptive data is presented, no statistical tests were conducted.

Supplementary Table 2. Between-group comparisons of plasma biomarkers in cohorts 1 (DDI), 2 (UGOT) and 3 (Biodegmar)

		A/T/N groups (n)				Statistical comparisons		
	Biomarker	A-/N-		A+/N-	A+/N+	*F* / t / *d* / η^2 ***(p)***	**A-/N-<br>*vs*<br>A+/N-**	**A-/N-<br>*vs*<br>A+/N+**
**Cohort 1 (DDI)**	**Plasma BD-tau**<br>Mean (SD)<br>[SD from ref-group]<br>**n**	CN 6.4 (6.8)<br>[0]<br>157	CN	6.7 (6.9)<br>[0.35]<br>22	14.2 (19.9)<br>[1.02]<br>42	*F*=10.7, η^2=.09,<br>(**<.001**)	n.s.	**<.001**
			MCI	7.1 (4.7)<br>[0.53]<br>23	11.4 (12.6)<br>[0.96]<br>120	*F*=17.7, η^2=.11,<br>(**<.001**)	n.s.	**<.001**
			All	6.9 (5.8)<br>[0.44]<br>45	12.1 (14.8)<br>[0.98]<br>162	*F*=20.5, η^2=.10,<br>(**<.001**)	n.s.	**<.001**
	**Plasma t-tau**<br>[SD from ref-group]<br>**n**	CN 9.1 (2.1)<br>[0]<br>157	CN	9.4 (2.7)<br>[0.21]<br>22	10.3 (2.6)<br>[0.67]<br>42	*F*=3.3, η^2=.03,<br>(**<.05**)	n.s.	**<.05**
			MCI	9.1 (2.2)<br>[0.23]<br>23	10.5 (2.4)<br>[0.77]<br>120	*F*=12.4, η^2=.08,<br>(**<.001**)	n.s.	**<.001**
			All	9.1 (2.4)<br>[0.22]<br>45	10.4 (2.4)<br>[0.75]<br>162	*F*=12.8, η^2=.07,<br>(**<.001**)	n.s.	**<.001**
	**Plasma NfL**<br>Mean (SD)<br>[SD from ref-group]<br>**n**	CN 20.5 (12.4)<br>[0]<br>157	CN	17.1 (6.1)<br>[0.10]<br>22	26.2 (15.1)<br>[0.84]<br>42	*F*=5.8, η^2=.05,<br>(**<.001**)	n.s.	**<.01**
			MCI	24.3 (10.4)<br>[0.72]<br>23	27.9 (13.7)<br>[0.93]<br>120	*F*=14.1, η^2=.09,<br>(**<.001**)	n.s.	**<.001**
			All	20.8 (9.3)<br>[0.42]<br>45	27.4 (14.0)<br>[0.91]<br>162	*F*=16.9, η^2=.09,<br>(**<.001**)	n.s.	**<.001**
**Cohort 2 (UGOT)**	**Serum BD-tau**<br>Mean (SD)<br>[SD from ref-group]<br>**n**	CN 1.9 (1.3)<br>[0]<br>9	Dementia	[b]	17.3 (6.9)<br>[3.73]<br>28	*t*=14.15, *d*=.5.4,<br>(**<.001**)	[a]	[a]
	**Serum t-tau**<br>Mean (SD)<br>[SD from ref-group]<br>**n**	CN 6.0 (8.6)<br>[0]<br>9		[b]	6.7 (10.1)<br>[0.67]<br>28	*t*=1.00, *d*=.0.4,<br>(n.s.)	[a]	[a]
**Cohort 3 (Biodegmar)**	**Serum BD-tau**<br>Mean (SD)<br>[SD from ref-group]<br>**n**	All 10.2 (6.6)<br>[0]<br>93	All	14.6 (7.9)<br>[1.35]<br>66	15.7 (13.3)<br>[1.43]<br>142	*F*=26.1, η^2=.15,<br>(**<.001**)	**<.001**	**<.001**
	**Plasma t-tau**<br>[SD from ref-group]<br>**n**	All 30.8 (14.1)<br>[0]<br>54	All	37.8 (14.4)<br>[0.98]<br>40	40.5 (18.4)<br>[1.11]<br>82	*F*= 8.3, η^2=.09,<br>(**<.001**)	**<.05**	**<.001**
	**Plasma NfL**<br>Mean (SD)<br>[SD from ref-group]<br>**n**	All<br>20.5 (10.9)<br>[0]<br>47	All	24.5 (13.1)<br>[0.65]<br>40	25.8 (16.7)<br>[0.69]<br>73	*F*=2.7, η^2=.03,<br>(n.s.)	n.s.	n.s.

Abbreviations: A+/-, positive or negative Cerebrospinal Fluid (CSF) marker for amyloid plaques; N+/-, positive or negative marker for neurodegeneration; CN, Cognitively Normal; MCI, Mild Cognitive Impairment; SD, standard deviation; n, number of cases; F, F statistic; t, t-test statistic; *d*= Cohen´s *d*; η^2, eta-squared; vs, versus; [a], no post-hoc comparisons performed; [b], no value; DDI, Dementia Disease Initiation cohort; UGOT, University of Gothenburg cohort; Biodegmar, Hospital del Mar, Barcelona memory clinic cohort. Please note that [SD from ref-group] shows the mean Z-score deviance from the reference group (A-/N-). The reference group Z-score is always kept as zero. Significant p-values (p<.05) are highlighted in bold. All statistical tests were two-sided and no adjustments for multiple comparisons were made.

Supplementary Table 3. Linear mixed models showing baseline plasma/serum biomarkers associations with longitudinal cognition (cohorts 1 and 3) and MRI (cohort 1)

	**Cognition (cohort 1, DDI)**		**MRI (cohort 1, DDI)**	**Cognition (cohort 3, Biodegmar)**	
**Response variables**	CERAD Recall	TMT-B	AD Meta ROI	MMSE	CDR
**Predictors**	*b* (95 % CI) [***p***]	*b* (95 % CI) [***p***]	*b* (95 % CI) [***p***]	*b* (95 % CI) [***p***]	*b* (95 % CI) [***p***]
Age	-0.30 (-0.39;-0.21) [**<.001**]	0.44 (0.35;0.52) [**<.001**]	-0.30 (-0.41;-0.19) [**<.001**]	-0.05 (-0.15;0.05) [.334]	0.04 (-0.01;0.09) [.092]
Education	0.18 (0.09;0.26) [**<.001**]	-0.19 (-0.27;-0.10) [**<.001**]		0.12 (0.02;0.23) [**<.05**]	-0.01 (-0.06;0.03) [.611]
Sex	0.15 (-0.03;0.33) [.099]		0.06 (-0.15;0.28) [.564]	-0.42 (-0.63;-0.21) **[< .001]**	0.10 (0.01;0.19) **[<.05]**
Plasma/serum BD-tau	-0.16 (-0.25;-0.06) (**<.001**)[a]	0.12 (0.03;0.21) (**<.01**)[a]	-0.05 (-0.17;0.06) (.350)[a]	-0.22 (-0.33;-0.12) **[< .001]**	0.10 (0.05;0.15) **[< .001]**
Years	-0.03 (-0.05;-0.01) (**<.05**)	0.03 (0.00;0.05) (**<.05**)	-0.06 (-0.11;-0.02) (**<.01**)	-0.27 (-0.34;-0.19) **[< .001)**	0.13 (0.11;0.16) **[< .001]**
BD-tau *Years	-0.05 (-0.05;0.00) (**<.05**)	0.04 (0.02;0.07) (**<.001**)	-0.06 (-0.10;-0.02) (**<.01**)	-0.12 (-0.21;-0.04) [**<.01**]	0.04 (0.01;0.07) [**<.01**]
Age	-0.33 (-0.43;-0.24) (**<.001**)	0.46 (0.37;0.54) (**<.001**)	-0.31 (-0.42;-0.20) (**<.001**)	-0.06 (-0.19;0.08) [.411]	0.01 (-0.07;0.06) [.792]
Education	0.17 (0.08;0.26) (**<.001**)	-0.18 (-0.27;-0.10) (**<.001**)		0.20 (0.06;0.34) [**<.01**]	-0.08 (-0.14;-0.02) [**<.05**]
Female sex	0.15 (-0.04;0.33) (.114)		0.05 (-0.17;0.27) (.679)	-0.33 (-0.60;-0.07) [**<.05**]	0.04 (-0.08; 0.16] [.530]
Plasma t-tau	-0.10 (-0.20;-0.01) (**<.05**)	0.07 (-0.02;0.16) (.122)	-0.08 (-0.20;0.03) (.166)	-0.17 (-0.31;-0.04) [**<.05**]	0.08 (0.01; 0.14) [**<.05**]
Years	-0.03 (-0.05;-0.01) (**<.01**)	0.03 (0.00;0.05) (**<.05**)	-0.06 (-0.11;-0.02) (**<.01**)	-0.22 (-0.30;-0.15) **[< .001]**	0.13 (0.10; 0.15) **[< .001]**
Plasma t-tau *Years	-0.03 (0.05;-0.01) (**<.01**)	0.01 (-0.01;0.04) (.189)	-0.01 (-0.04;0.03) (.722)	-0.04 (-0.11;0.04) [.356]	0.02 (-0.002; 0.05) [.068]
Age	-0.28 (-0.38;-0.17) (**<.001**)	0.41 (0.32;0.51) (**<.001**)	-0.27 (-0.39;-0.14) (**<.001**)	0.04 (-0.11;0.18) [.614]	-0.02 (-0.08;0.05) [.639]
Years of Education	0.18 (0.09;0.27) (**<.001**)	-0.18 (-0.27;-0.10) (**<.001**)		0.26 (0.12;0.40) **[< .001]**	-0.09 (-0.15;-0.03) [**<.01**]
Sex	0.14 (-0.05;0.32) (.158)		0.02 (-0.21;0.24) (.884)	-0.24 (-0.51;0.03) [.077]	0.01 (-0.11; 0.13) [.896]
Plasma NfL	-0.09 (-0.19;-0.02) (.108)	0.06 (-0.03;0.16) (.200)	-0.06 (-0.19;0.07) (.342)	-0.24 (-0.39;-0.09) **[<.01]**	0.15 (0.08; 0.22) **[<.001]**
Years	-0.03 (-0.05;-0.01) (**<.01**)	0.03 (0.01;0.05) (**<.05**)	-0.07 (-0.11;-0.02) (**<.01**)	-0.23 (-0.31;-0.16) **[<.001]**	0.12 (0.10; 0.15) **[< .001]**
Plasma NfL *Years	-0.03 (0.05;0.00) (**<.05**)	0.03 (0.01;0.06) (**<.05**)	-0.05 (-0.10;0.00) (.053)	-0.11 (-0.19;-0.03) **[<.01]**	0.02 (-0.01; 0.05) [.190]

Abbreviations. *b*, beta-coefficient; CI, Confidence Interval; p, p-value; BD-tau, Brain-Derived tau; t-tau, total-tau; NfL, Neurofilament Light Chain; DDI, Dementia Disease Initation; Biodegmar, Hospital del Mar, Barcelona memory clinic cohort. Please note that BD-tau was measured in plasma for cohort 1 and in serum for cohort 3. Significant p-values (p<.05) are highlighted in bold. All statistical tests were two-sided and no adjustments for multiple comparisons were made.

Supplementary Table 4. ROC analyses of plasma p181 and plasma (cohort 1, DDI) or serum (cohort 3, Biodegmar) BD-tau.

	**Standard of truth (CSF markers)**	**Measure**	**AUC (95 % CI)**	**Cases/ controls**	**Spec.**	**Sens.**	**Cut-off**
DDI	CN A-/N- vs MCI A+/N+	Plasma p-tau181	.957 (.903-1)	118/156	.846	.712	≥11.96
	CN A-/N- vs MCI A+/N+	Plasma BD-tau	.700 (.638-.761)	118/156	.592	.717	≥5.31
Biodegmar	All A-/N- vs All A+/N+	Plasma p-tau181	0.817 (.730-.905)	61/40	.675	.853	≥12.55
	All A-/N- vs All A+/N+	Serum BD-tau	0.762 (.659-.865)	61/40	.725	.820	≥9.83

Abbreviations. A+/-, positive or negative Cerebrospinal Fluid (CSF) marker for amyloid plaques; N+/-, positive or negative marker for neurodegeneration; BD-tau, Brain-Derived tau; p-tau; phosphorylated tau; CN, Cognitively Normal; MCI, Mild Cognitive Impairment; AUC, Area Under curve; CI, confidence interval; Spec., Specificity; Sens., Sensitivity; DDI, Dementia Disease Initiation cohort; Biodegmar, Hospital del Mar, Barcelona memory clinic cohort. Cut-offs were determined using the Youden index.

Supplementary Table 5. Linear mixed models showing A/N groups based on plasma/serum (blood) biomarkers and their associations with longitudinal cognition (cohorts 1 and 3) and MRI (cohort 1)

**Response variables**	**DDI Cognition**		**DDI MRI**	**Biodegmar Cognition**	
	CERAD Recall	TMT-B	AD Meta ROI	MMSE	CDR
**Predictors**	*b* (95 % CI) [***p***]	*b* (95 % CI) [***p***]	*b* (95 % CI) [***p***]	*b* (95 % CI) [***p***]	*b* (95 % CI) [***p***]
Age	-0.24 (-0.33;-0.15) [**<.001**]	0.39 (0.31;0.48) [**<.001**]	-0.25 (-0.37;-0.14) [**<.001**]	-0.06 (-0.23;0.11) [.492]	0.02 (-0.05;0.09) [.513]
Education	0.18 (0.10;0.27) [**<.001**]	-0.19 (-0.27;-0.11) [**<.001**]		0.18 (0.02;0.33) **[<.05]**	-0.04 (-0.11;0.03) [.223]
Female Sex	0.11 (-0.07;0.28) [.224]		0.03 (-0.19;0.24) [.821]	-0.52 (-0.83;-0.21) **[<.01]**	0.08 (-0.05;0.21) [.233]
Years	0.01 (-0.03;0.04) [.652]	-0.01 (-0.04;0.04) [.841]	-0.01 (-0.08;0.07) [.911]	-0.17 (-0.34; -0.01) **[<.05]**	0.08 (0.02;0.14) **[<.01]**
Blood A+/N-	-0.59 (-0.91;-0.28) (**<.001**)	0.22 (-0.01;0.53) (.178)	-0.19 (-0.61;0.23) (.368)	-0.05 (-0.57; 0.47) [.851]	0.10 (-0.12;0.33) [.369]
Blood A+/N+	-0.69 (-0.91;-0.47) (**<.001**)	0.50 (0.28;0.71) (**<.001**)	-0.41 (-0.70;-0.13) (**<.01**)	-0.65 (-1.06;-0.23) **[<.01]**	0.38 (0.20;0.56) **[<.001]**
Blood A-/N+	-0.09 (-0.32;0.15) (.481)	-0.01 (-0.24;0.23) (.971)	-0.09 (-0.39;0.21) (.540)	-0.19 (-0.78;0.41) [.538]	0.24 (-0.02;0.50) [.070]
Blood A+/N-*Years	-0.05 (-0.13;0.03) (.221)	0.02 (-0.06;0.11) (.599)	0.04 (-0.13;0.20) (.652)	-0.03 (-0.28;0.22) [.813]	0.10 (0.02 ; 0.19) **[<.05]**
Blood A+/N+*Years	-0.08 (-0.14;-0.03) (**<.01**)	0.08 (0.02;0.14) (**<.01**)	-0.15 (-0.26;-0.05) (**<.01**)	-0.12 (-0.32;0.08) [.255]	0.07 (0.01;0.14) **[<.05]**
Blood A-/N+*Years	-0.04 (-0.10;0.02) (.199)	0.01 (-0.05;0.7) (.698)	-0.05 (-0.16;0.06) (.363)	-0.10 (-0.35;0.16) [.457]	0.04 (-0.06;0.13) [.474]

Abbreviations. A+/-, positive or negative Plasma p181 marker; N+/-, positive or negative Plasma (cohort 1, DDI) or serum (cohort 3, Biodegmar) BD-tau marker; *b,* beta-coefficient; CI, Confidence Interval; p, p-value; DDI, Dementia Disease Initiation cohort; Biodegmar, Hospital del Mar, Barcelona memory clinic cohort. Significant p-values (p<.05) are highlighted in bold. All statistical tests were two-sided and no adjustments for multiple comparisons were made.

Supplementary Table 6. Between-group comparisons of CSF biomarkers in cohorts 1 and 3

	Biomarker	A/T/N groups (n)				Statistical tests		
		**A-/N-**		**A+/N-**	**A+/N+**	*F / η^2* ***(p)***	**A-/N- *vs* A+/N-**	**A-/N- *vs* A+/N+**
**Cohort 1 (DDI)**	**CSF BD-tau** Mean (SD) [SD from ref-group]	CN 241.5 (80.5) [0]	CN	262.2 (96.4) [0.84] 22	609.8 (291.1) [2.24] 42	*F*=92.0, η^2=.47, (**<.001**)	n.s.	**<.001**
			MCI	297.1 (72.9) [1.13] 23	641.9 (280.5) [2.31] 120	*F*=203.3, η^2=.58, (**<.001**)	**<.01**	**<.001**
			All	280.0 (86.1) [0.99] 45	633.6 (282.7) [2.29] 162	*F*=237.7, η^2=.57, (**<.001**)	**<.05**	**<.001**
	**CSF NfL** Mean (SD) [SD from ref-group]	CN 2551.7 (1425.6) [0]	CN	2661.0 (736.4) [0.78] 22	4324.6 (2386.9) [1.58] 42	*F*=23.3, η^2=.18, (**<.001**)	n.s.	**<.001**
			MCI	3271.3 (1256.8) [1.09] 23	4448.1 (2266.5) [1.67] 120	*F*=57.8, η^2=.28, (**<.001**)	**<.01**	**<.001**
			All	2972.9 (1070.5) [0.94] 45	4416.1 (2291.5) [1.65] 160	*F*=67.2, η^2=.27, (**<.001**)	**<.05**	**<.001**
**Cohort 3 (Biodegmar)**	**CSF BD-tau** Mean (SD) [SD from ref-group] **n**	All 263.1 (108.9) [0] 108	All	354.9 (108.1) [1.61] 78	660.1 (264.3) [2.70] 177	*F*=253.5, η^2=.59, (**<.001**)	**<.001**	**<.001**

Abbreviations: A+/-, positive or negative Cerebrospinal Fluid (CSF) marker for amyloid plaques; N+/-, positive or negative marker for neurodegeneration; CN, Cognitively Normal; MCI, Mild Cognitive Impairment; SD, standard deviation; n, number of cases; F, F statistic; η^2, eta-squared; vs, versus; DDI, Dementia Disease Initiation cohort; Biodegmar, Hospital del Mar, Barcelona memory clinic cohort. Please note that [SD from ref-group] shows the mean Z-score deviance from the reference group (A-/N-). The reference group Z-score is this always kept as zero. Significant p-values (p<.05) are highlighted in bold. All statistical tests were two-sided and no adjustments for multiple comparisons were made.

Supplementary Table 7. Linear mixed models showing baseline CSF biomarkers associations with longitudinal cognition (cohorts 1 and 3) and MRI (cohort 1)

	**Cognition (cohort 1, DDI)**		**MRI (cohort 1, DDI)**	**Cognition (cohort 3, Biodegmar)**	
**Response variables**	CERAD Recall	TMT-B	AD Meta ROI	MMSE	CDR
**Predictors**	*b* (95 % CI) [*p*]	*b* (95 % CI) [*p*]	*b* (95 % CI) [*p*]	*b* (95 % CI) [*p*]	*b* (95 % CI) [*p*]
Age	-0.18 (-0.27;-0.09) [**<.001**]	0.37 (0.28;0.46) [**<.001**]	-0.25 (-0.37;-0.13) [**<.001**]	-0.03 (-0.12;0.06) [.549]	0.02 (-0.03;0.06) [.435]
Education	0.14 (0.06;0.22) [**<.001**]	-0.17 (-0.25;-0.08) [**<.001**]		0.18 (0.09;0.28) [**<.001**]	-0.05 (-0.09;-0.002) **[<.05]**
Female Sex	0.11 (-0.06;0.28) [.187]		0.04 (-0.18;0.26) [.736]	-0.25 (-0.44;-0.06) **[<.05]**	0.04 (-0.04;0.13) [.328]
CSF BD-tau	-0.39 (-0.49;-0.30) (**<.001**)	0.18 (0.08;0.27) (**<.001**)	-0.10 (-0.23;0.02) (.108)	-0.08 (-0.18;0.02) [.121]	0.09 (0.04;0.13) [**<.001**]
Years	-0.03 (-0.06;-0.01) (**<.01**)	0.03 (0.01;0.06) (**<.01**)	-0.07 (-0.11;-0.03) (**<.01**)	-0.23 (-0.30;-0.15) [**<.001**]	0.13 (0.11;0.15) [**<.001**]
CSF BD-tau *Years	-0.05 (-0.07;-0.02) (**<.001**)	0.05 (0.03;0.08) (**<.001**)	-0.05 (-0.09;-0.01) (**<.05**)	-0.16 (-0.24;-0.08) [**<.001**]	0.01 (-0.01;0.04) [.247]
Age	-0.15 (-0.24;-0.06) [**<.001**]	0.33 (0.24;0.42) (**<.001**)	-0.23 (-0.35;-0.11) [**<.001**]	-0.04 (-0.13;0.05) [.346]	0.02 (-0.02;0.06) [.334]
Education	0.14 (0.06;0.22) [**<.001**]	-0.16 (-0.24;-0.08) (**<.001**)		0.19 (0.09;0.28) [**<.001**]	-0.05 (-0.09;-0.01) **[<.05]**
Female sex	0.13 (-0.04;0.29) [.137]		0.04 (-0.18;0.25) [.719]	-0.22 (-0.41;-0.03) **[<.05]**	0.03 (-0.06;0.11) [.575]
CSF t-tau	-0.44 (-0.53;-0.34) (**<.001**)	0.24 (-0.15;0.34) (**<.001**)	-0.12 (-0.24;-0.01) (**<.05**)	-0.08 (-0.18;0.02) [.122]	0.08 (0.04;0.13) [**<.001**]
Years	-0.04 (-0.06;-0.01) (**<.01**)	0.03 (0.01;0.06) (**<.01**)	-0.07 (-0.11;-0.02) (**<.01**)	-0.23 (-0.30;-0.16) [**<.001**]	0.13 (0.11;0.15) [**<.001**]
CSF t-tau *Years	-0.04 (-0.07;-0.02) (**<.001**)	0.05 (0.03;0.08) (<.001)	-0.06 (-0.11;-0.02) (**<.01**)	-0.17 (-0.25;-0.10) [**<.001**]	0.03 (0.01;0.05) **[<.05]**
Age	-0.14 (-0.25;-0.04) (**<.01**)	0.33 (0.23;0.43) (**<.001**)	-0.26 (-0.39;-0.13) (**<.001**)		
Years of Education	0.16 (0.08;0.25) (**<.001**)	-0.17 (-0.26;-0.09) (**<.001**)			
Female Sex	0.03 (-0.15;0.21) (.754)		0.03 (-0.20;0.26) (.812)		
CSF NfL	-0.31 (-0.42;-0.21) (**<.001**)	0.19 (-0.08;0.29) (**<.001**)	-0.07 (-0.21;0.07) (.305)		
Years	-0.04 (-0.06;-0.01) (**<.01**)	0.03 (0.01;0.06) (**<.05**)	-0.07 (-0.11;-0.03) (**<.01**)		
CSF NfL *Years	-0.03 (0.06;-0.01) (**<.01**)	0.04 (0.01;0.07) (**<.01**)	-0.03 (-0.08;0.01) (.150)		

Abbreviations. *b,* beta-coefficient; CI, Confidence Interval; p, p-value; CSF, Cerebrospinal Fluid; BD-tau, Brain-Derived tau; t-tau, total-tau; NfL, Neurofilament Light Chain; DDI, Dementia Disease Initiation cohort; Biodegmar, Hospital del Mar, Barcelona memory clinic cohort. Significant p-values ($p<.05$) are highlighted in bold. All statistical tests were two-sided and no adjustments for multiple comparisons were made.

Supplementary Figure 1. Forest plots showing plasma and CSF biomarker levels according to A/N classification in cohort 1 (DDI).

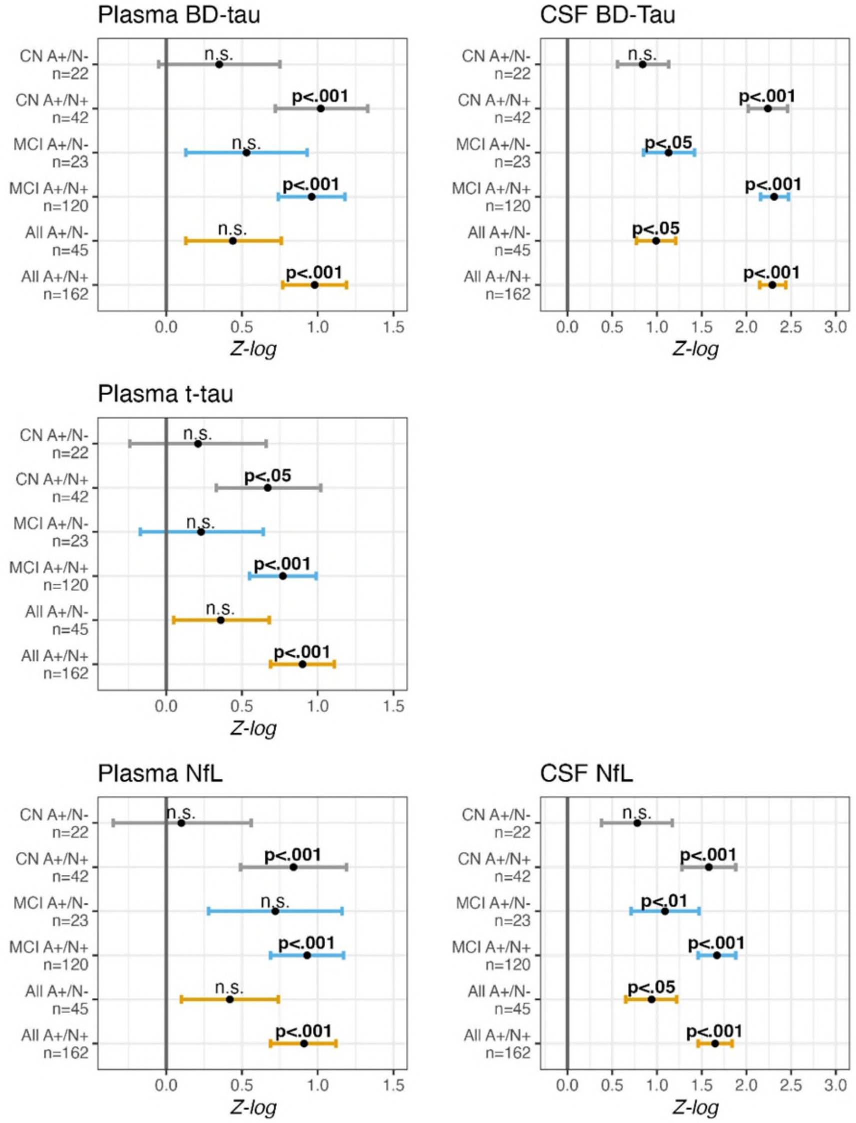

This forest plot displays the mean elevation of blood-based BD-tau according to amyloid (A) and neurodegeneration (N) status in the Alzheimer's Disease (AD) continuum across cohorts 1 through 3. Point estimates are log-transformed and standardized (Z-log), expressed as standard deviations relative to A-/N- cases (represented by a grey vertical bar, normalized to mean 0 as the reference). Error bars denote 95% confidence intervals. Groups are split by Cognitively Normal (CN) and Mild Cognitive Impairment (MCI) cases within cohort 1 (Dementia Disease Initiation (DDI) cohort). All statistical tests were two-sided and unadjusted for multiple comparisons.

Supplementary Figure 2. Correlations of CSF BD-tau and NfL with t-tau in cohorts 1 (DDI) and 3 (Biodegmar)

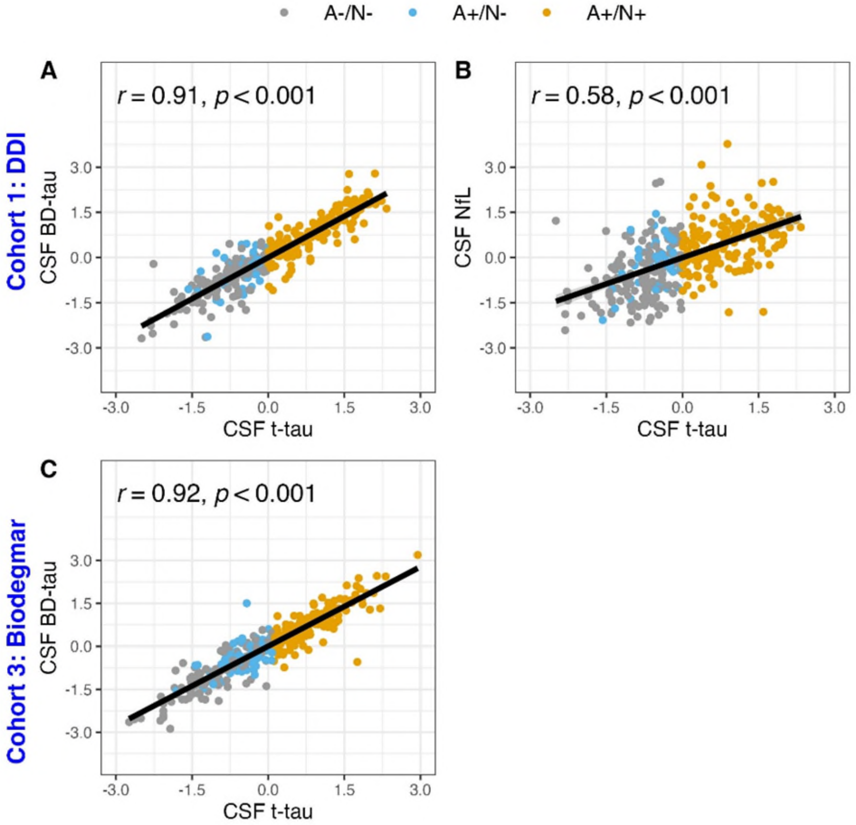

Pearsons correlations between Z-log Cerebrospinal Fluid (CSF) total tau (t-tau) and CSF neurodegeneration markers. Panels **A-B** shows correlations to CSF Brain Derived tau (BD-tau) and Neurofilament Light Chain (NfL) in cohort 1 (Dementia Disease Initiation (DDI) cohort). Panel **C** shows the correlation between CSF t-tau and CSF BD-tau within cohort 3 (Biodegmar, Hospiral del Mar, Barcelona memory clinic cohort). All statistical tests were two-sided, and unadjusted for multiple comparisons.

Supplementary Figure 3. Correlation of plasma/serum neurodegeneration biomarkers with CSF t-tau in cohorts 1 (DDI), 2 (UGOT), and 3 (Biodegmar).

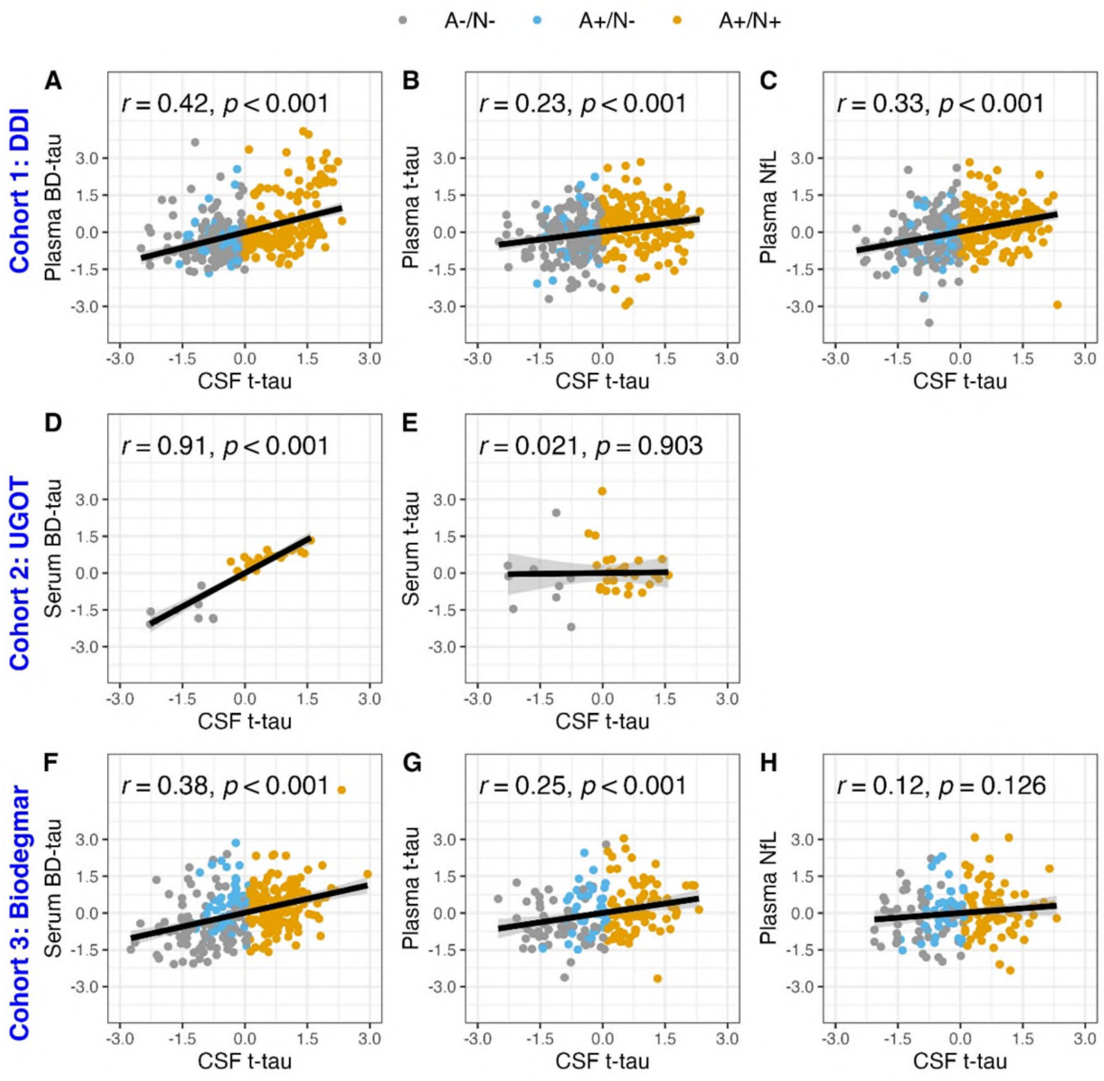

Pearsons correlations between Z-log Cerebrospinal Fluid (CSF) total tau (t-tau) to blood-based neurodegeneration markers. Panels **A-C** show correlations to plasma Brain Derived tau (BD-tau), total-tau (t-tau) and Neurofilament Light Chain (NfL) within cohort 1 (Dementia Disease Initiation (DDI) cohort). Panels **D-E** shows CSF t-tau correlations to serum BD-tau and serum t-tau within cohort 2 (University of Gothenburg (UGOT) cohort). Panels **F-G** shows correlations between CSF t-tau and serum BD-tau, plasma t-tau and plasma NfL within cohort 3 (Biodegmar, Hospiral del Mar, Barcelona memory clinic cohort). All statistical tests were two-sided, and unadjusted for multiple comparisons.

Supplementary Figure 4. Comparison of plasma/serum BD-tau in AD versus non-AD groups in cohorts 1 (DDI) and 3 (Biodegmar).

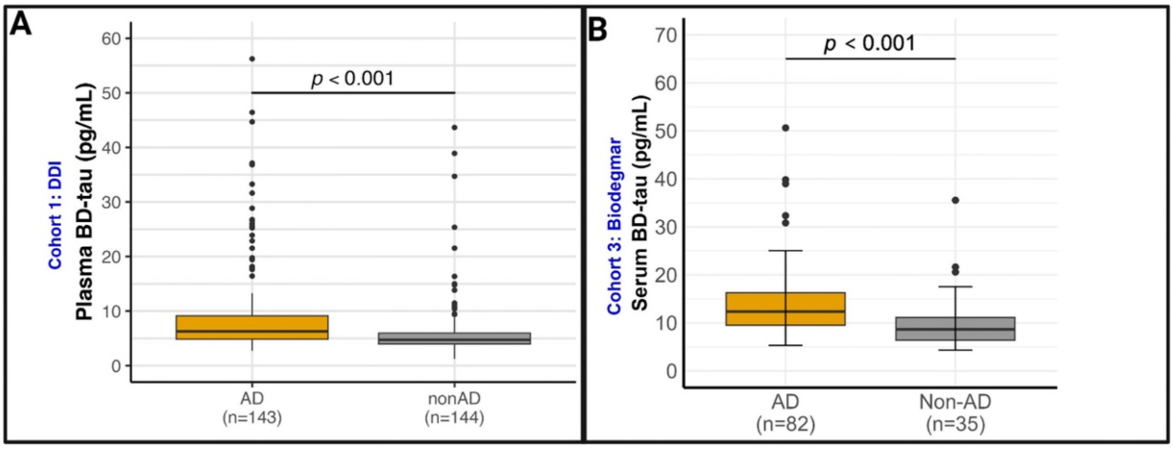

Panel **A** shows median plasma Brain-Derived tau (BD-tau) differences (black horizontal bar within each box) between AD MCI and non-AD MCI cases within cohort 1 (Dementia Disease Initiation (DDI) cohort). Panel **B** shows median serum Brain-Derived tau (BD-tau) differences between cognitively impaired (mixed MCI and dementia) AD and non-AD cases within cohort 3 (Biodegmar, Hospital del Mar, Barcelona memory clinic cohort). Comparisons between groups were performed with independent samples t-tests. The statistical tests were two-sided
